# Comparative Analysis of Four Buckwheat Species Based on Morphology and Complete Chloroplast Genome Sequences

**DOI:** 10.1038/s41598-017-06638-6

**Published:** 2017-07-26

**Authors:** Cheng-Long Wang, Meng-Qi Ding, Chen-Yan Zou, Xue-Mei Zhu, Yu Tang, Mei-Liang Zhou, Ji-Rong Shao

**Affiliations:** 1 School of Life Sciences, Sichuan Agricultural University, Yaan, Sichuan 625014 China; 2 School of Resources and Environment, Sichuan Agricultural University, Chengdu, Sichuan 611130 China; 3 Department of Tourism Culture, Sichuan Higher Institute of Cuisine, Chengdu, Sichuan 610072 China; 4 Biotechnology Research Institute, Chinese Academy of Agricultural Sciences, Beijing, 100081 China

## Abstract

Buckwheat is a nutritional and economically crop belonging to *Polygonaceae*, *Fagopyrum*. To better understand the mutation patterns and evolution trend in the chloroplast (cp) genome of buckwheat, and found sufficient number of variable regions to explore the phylogenetic relationships of this genus, two complete cp genomes of buckwheat including *Fagopyrum dibotry*s (*F*. *dibotrys*) and *Fagopyrum luojishanense* (*F*. *luojishanense*) were sequenced, and other two *Fagopyrum* cp genomes were used for comparative analysis. After morphological analysis, the main difference among these buckwheat were height, leaf shape, seeds and flower type. *F*. *luojishanense* was distinguishable from the cultivated species easily. Although the *F*. *dibotrys* and two cultivated species has some similarity, they different in habit and component contents. The cp genome of *F*. *dibotrys* was 159,320 bp while the *F*. *luojishanense* was 159,265 bp. 48 and 61 SSRs were found in *F*. *dibotrys* and *F*. *luojishanense* respectively. Meanwhile, 10 highly variable regions among these buckwheat species were located precisely. The phylogenetic relationships among four *Fagopyrum* species based on complete cp genomes was showed. The results suggested that *F*. *dibotrys* is more closely related to *Fagopyrum tataricum*. These data provided valuable genetic information for *Fagopyrum* species identification, taxonomy, phylogenetic study and molecular breeding.

## Introduction

Buckwheat is an ancient dicotyledonous crop belonging to *Polygonaceae*, *Fagopyrum*, which is widely distributed around the world^1^. The *Fagopyrum* was classified into 25 species, which including two recently identified wild species *F*. *luojishanense* and *F*. *hailuogouense* ^2, 3^. Buckwheat is a kind of traditional and economically food crop in Southwest China with the advantages of short growth period, high adaptability, high yield and resistance to harsh environments where the major food crops may hard to survive. At the same time, buckwheat also known for their high-quality proteins and rich in many rare components including flavonoids and phytosterols which have healing effects on some chronic diseases^4^ and play an important role in anti-oxidation metabolism. Due to its high content of starch, microelement, lysine and methionine, buckwheat also has been paid attention to its feeding value in recent years. At present, the potential commercial values of buckwheat have been widely paid attention. It was difficult for the buckwheat species identification, especially for the wild buckwheat, which impede the effective exploration and utilization of the resource advantage. Morphological research is the foundation of classification and evolution research. Meanwhile, the biological components and nutritions of plants which influenced by weather and metabolism are normally different among species. Based on morphological characteristics, the *Polygonaceae* family which is morphologically diverse and distributed was divided into two classical subfamilies, *Polygonoideae Eaton* and *Eriogonoideae Arn* ^5^. It has been supported by molecular phylogenies with a broad sampling of genera^6^.

During the past years, the use of cp DNA sequence data has been proved useful for phylogenetic relationships and inferring phylogenetic relationships in genus *Fagopyrum* at taxonomic levels. Ohnishi *et al*. classified 11 species of *Fagopyrum* based on their morphology, isozyme variability and RFLP of cp DNA^7^. After that, Yasui *et al*. analyzed the 3′ of *rbcL*, the *accD* 5′ end regions and the ITS regions of 12 species of *Fagopyrum*, they found the polyploidization in *F*. *cymosum* might have occurred more than once^8^. On the other hand, Ohsako *et al*. studied the intra- and interspecific phylogeny of *Fagopyrum* using *trnC-rpoB*, and *trnK*/*matK* in the urophyllum group, and results showed that these two regions can be used to study genetic relationships at lower taxonomic levels^9^. The maturase K (*matK*) gene of the chloroplast genome has been provided good resolution in phylogenetic analysis of several groups within genus *Fagopyrum*. In 2001, Ohsako *et al*. used *trnK*/*matK* regions of cp DNA to confirm the phylogenetic relationship of two wild *Fagopyrum* (*Polygonaceae*) species^10^. Two years later, Nishimoto *et al*. used two nucleotide genes (*FLO*/*LFY* and *AG*) and three segments of cp DNA to analyze the species of *Fagopyrum*, however their results showed that the topologies were partially incongruent between the nuclear and chloroplast DNA phylogenies^11^. All of these reports mentioned above demonstrated that cp DNA analysis is useful in *Fagopyrum* classification, but they only focused on some parts of the chloroplast genome. It became a major problem, only using some portions of the chloroplast genome for phylogenetic research, the incomplete dates will limit the development of plant evolutionary studies^12^. Owing to the incomplete sampling, insufficient phylogenetic markers and complex evolutionary issues, the phylogenetic relationships among *Fagopyrum* species are still not fully understood.

Chloroplast genome sequences could provide sufficient information for analysis and comparison the diversifications among plant species. It contains a series of function genes which play a key role in plant cells, especially orchestrate the photosynthesis process, sugar synthesizing and certain fixation^12^. On the other hand, the chloroplast DNA also has the high copy number which makes it could be easily extracted from samples. The chloroplast sequence is more conserved than nuclear sequence^13^, the non-recombinant and mostly uniparental inheritance characteristics of the chloroplast genome makes the gene content and genome structure is highly conservative in plants^14^. But it also exhibit different mutation events^15^, which will provide many sequences divergence information between species and it also could been used as a potential tool for evolutionary, taxonomy, phylogeny studies in plants^16–19^. All in all, the sequencing data of chloroplast DNA could be applied to DNA barcoding^20^, phylogeny reconstruction^21^ and transplastomic studies^22^.

Our research group has devoted to the collection of buckwheat germplasm resources for decades. *F*. *luojishanense* is a new wild buckwheat specie which was discovered recently^2^. It is a transitional species of buckwheat, both phylogenetic and genomic studies are lacking for this species. While the *F*. *dibotrys* belongs to *Fagopyrum* genus, which has been used as herb-medicine for many years, but the affinity of *F*. *dibotrys* also remain debatable and the relationship between *F*. *dibotrys* and other two cultivated buckwheat (*F*. *esculentum* and *F*. *tataricum*) was still controversy. To discuss the clear taxonomic status, additional sequence information such as chloroplast genome sequence is highly desirable. Until recently, there are only two complete chloroplast genome of buckwheat was sequenced and reported^23, 24^, which is insufficient for discussing the evolutionary relationships among buckwheat species in many cases. At the same time, the reported biomarkers like ITS and *matK* sequences could not to reveal the phylogenetic relationships in this genus perfectly and resolve the species identification problems accurately. Meanwhile, the results of different molecular markers also have considerable differences. The more important is the sequence of wild buckwheat chloroplast genomes not only could be utilized for its phylogenetic studies but also could provide more useful information for other practical aspects. For example, we can highlight the physiologically important traits genes in buckwheat^25^ and developing species specific transformation vectors^26^ through analyse the chloroplast genome sequence. All in all, the research of the buckwheat chloroplast genome sequence opens an avenue for the application of buckwheat plastid genetic engineering. And it is prerequisite for an efficient breeding program that assessing genetic variability within *Fagopyrum* and its variation among populations.

In the present study, we compared cultivated buckwheat and wild buckwheat in morphology aspect. At the same time, we sequenced chloroplast genomes of two wild buckwheat species and comparative analyzed four complete chloroplast genomes including cultivated buckwheat species. After that, we also discussed the phylogenetic relationship between the new species and some other species. Finally, we tried to answer the following questions: (1) Are the differences among buckwheat in morphology related with the chloroplast genomes evolution? (2) What is the typical structural pattern of chloroplast genomes in buckwheat? (3) What kind of mutation events happened among the four chloroplast genomes? (4) Identified highly variable regions by compared the four complete chloroplast genomes, which could be utilized as potential markers or candidate DNA barcoding for phylogenetic analysis. To our knowledge, this paper is the first report of different wild buckwheat’s complete chloroplast genome sequences and it is also the first comprehensive analysis based on complete chloroplast genomes for wild buckwheat with cultivated buckwheat. Using the *Fagopyrum* chloroplast genome sequences for the phylogenetic analysis could demonstrate the evolution of each species and provide abundant information for potential biomarkers to the species identification, taxonomy, phylogenetic research and assist in utilization and exploration of wild buckwheat.

## Results

### Morphological Analysis

**Figure 1:**
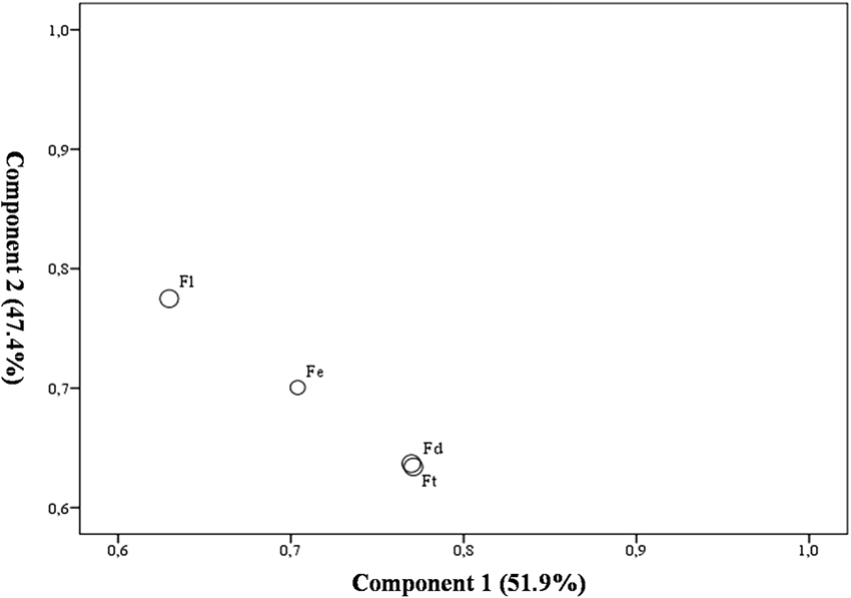
Scatter plot of buckwheat based on two discriminate components of PCA. The Ft represent *F. tataricum*, Fe represent *F. esculentum*, Fd represent *F. dibotrys* and the Fl represent *F. luojishanense*.

Phylogenetic relationships based on morphological characteristics were discussed in this section. The morphological characteristics of different buckwheat species indicated that the differences among four buckwheat including plant height, stem thickness, node number, pistil number, inflorescence, seeds luster and so on. Results reflected the morphological differences of four buckwheat species were shown in Table 1. On the other hand, *Fagopyrum* species contained a variety of nutrients like flavonoids and amino acids. In the present study, we also measured the agronomy data of different buckwheat, such as thousand grain weight, protein, amino acid, flavonoids content and so on, which were showed in Supplementary Table S1. The values represent means of different independent replicates ± SE, respectively. From the results, the morphological characteristics of wild buckwheat species were prominently different from the cultivated species. Meanwhile, the amino acid content of the four different buckwheat was basically different, and the flavonoids in *F*. *dibotrys* and *F*. *tataricum* were higher than other two species. At the same time, in order to further determine and evaluate the differences of four *Fagopyrum* species, eight indicators were processed by principal component analysis (PCA). The scatter plot constructed by two discriminate components based on PCA was shown in Fig. 1. The components 1 explained 51.9% of the variance, and components 2 explained 47.4%. It shown that the wild buckwheat *F*. *luojishanense* was easily distinguished from other three buckwheat, but it still hard to discriminate the other three buckwheat species clearly. In summary, the PCA result based on combining the morphology data and agronomy data could not well determine the differences among the four buckwheat species. The relationships of these four species were still unclear when only using morphological and agronomy characteristics data. Therefore, other analysis should be used to obtain better and reliable results. And these differences probable derived from the chloroplast genome of buckwheat, and this hypothesis also need further comparative analysis of complete chloroplast genome sequences.

**Table 1:** Morphological description of four *Fagopyrum* species.

Index	*F. tataricum*	*F. esculentum*	*F. dibotrys*	*F. luojishanense*
Origin	Cultivar	Cultivar	Wild	Wild
Height (cm)	55.03 ± 1.44	62.9 ± 0.72	69.93 ± 0.85	54.37 ± 1.54
Plant Type	erect	erect	mostly erect, sometimes semi-erect	mostly erect, sometimes grovel
Stem color	green	green or red	red-brown	red-brown
Stem thick (cm)	0.37 ± 0.01	0.38 ± 0.01	0.31 ± 0.01	0.33 ± 0.01
Leaf	Leaves alternate, rugulose and small postulate on the surface, triangular or wide-triangular, (0.9–)3–6.5 cm long and (0.7–)1.6–5 cm wide	Leaves alternate, rugulose and small postulate on the surface, triangular or ovate-triangular, (0.8–)2.5–7 cm long and (0.6–)2–5 cm wide	Leaves alternate, densely pubescent in the two surfaces, rugulose and small postulate on the surface, leaf blade triangular, (0.9–)4–12 cm long and (0.5–)3–11 cm wide	Leaves alternate, no pubescent in the two surfaces, ovate-triangular, (0.6–)1.7–6 cm long and (0.7–)1.2–5.1 cm wide
Petioles	Leaves in the base of stem have long petioles, leaves in upper part of stem have small and short petioles	Leaves in the base of stem have long petioles, leaves in upper part of stem have no petioles or short petioles	Leaves in the base of stem have long petioles, leaves in upper part of stem have no petioles or short petioles	Petioles of the base leaves as long as base leaves, leaves in upper part of stem have shorter petioles or no petioles
Inflorescence type	Racemose inflorescences, axillary and terminal	Capitate and racemose inflorescences, axillary and terminal	Capitate inflorescences, axillary and terminal	Racemose inflorescences, axillary and terminal
Peduncle	There have nodes in the middle of peduncle	There have no nodes in the peduncle	There have nodes in the middle of peduncle	There have no nodes in the peduncle
Number of Perianth	Perianth 5, white or pinked	Perianth 5, white or pinked	Perianth 5, white or pinked	Perianth 5, white or pink red
Number of Stamens	8	8	8	8
Number of style	3	3	3	3
Flower type^#^	Hetero-type flower	Hetero-type flower	Same-type flower	Same-type flower
Seed color	Black-brown, no lustrous	Dark-brown, no lustrous	Black-brown, no lustrous	Brown, lustrous
Seed shape	Achene, Long ovate	Achene, Ovate	Achene, Broadly ovate	Achene, Ovate
Winged seed	No winged	No winged	No winged	Winged

^#^The same-type flower: Flower has different length of pistil and stamen. The hetero-type flower: The pistil length of flower is equal to stamen.

### Chloroplast Genome Features of *F*. *dibotrys* and *F*. *luojishanense*

**Figure 2:**
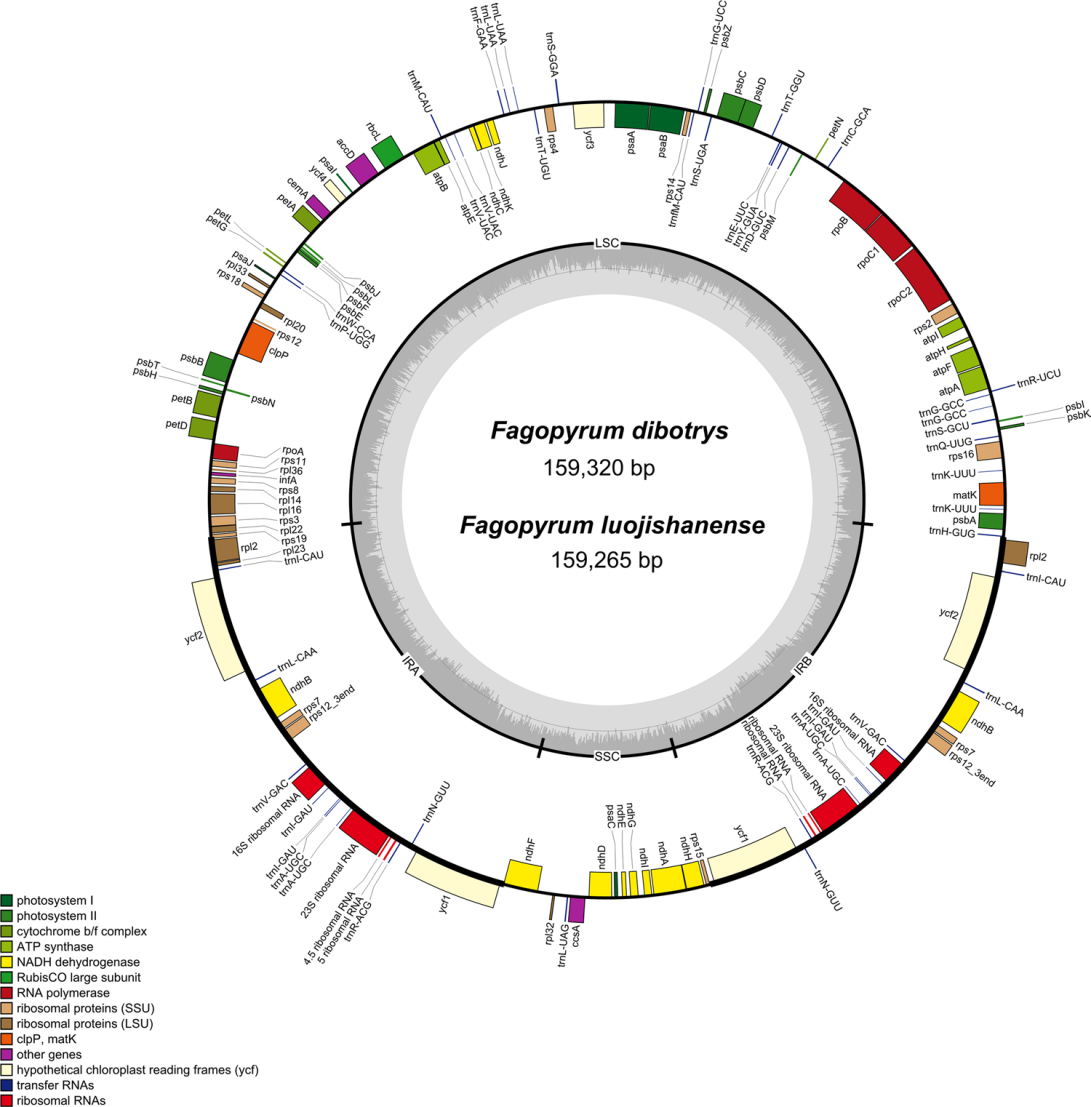
Gene map of *Fagopyrum loujishanenes* and *Fagopyrum dibotrys*. The annotation of the genome was performed using DOGMA. The genes shown outside of the circle are transcribed clockwise, while those inside are counterclockwise.

In this paper, the chloroplast genomes of two wild buckwheat species were sequenced using the Illumina HiSeq. 4000 system. The *F*. *luojishanense* and *F*. *dibotrys* produced 17,562,298 and 29,833,124 paired-end raw reads after sequencing. In these paired-end reads, 203,148 and 1,154,010 chloroplast genome reads were extracted after aligned with the reference genomes, and the content of chloroplast reads was 1.16% and 3.87%. The coverage of *F*. *luojishanense* and *F*. *dibotrys* chloroplast genomes were 191 and 1086 respectively. The entire chloroplast genome of *F*. *luojishanense* consisted of 159,265 bp nucleotides and the chloroplast genome of *F*. *dibotrys* had a length of 159,320 bp. The map of gene was shown in Fig. 2. The results of comparison of the four complete chloroplast genomes contents were shown in Table 2. The *Fagopyrum* chloroplast genomes include a pair of IR regions of 30,870 bp in *F*. *luojishanense* and 30,817 bp in *F*. *dibotrys*. And it was separated by a LSC region of 84,431 bp in *F*. *luojishanense* and 84,422 bp in *F*. *dibotrys* and a SSC region of 13,094 bp in *F*. *luojishanense* and 13,264 bp in *F*. *dibotrys*. The average GC content was 37.8% in *F*. *luojishanense* and 37.9% in *F*. *dibotrys* respectively, which was almost similar value with each other among the four complete *Fagopyrum* chloroplast genomes. Both of the two *Fagopyrum* chloroplast genomes contained 114 different functional genes, including 81 protein-coding genes, 29 tRNA genes, and 4 rRNA genes, which were identical to those of other published *Fagopyrum* chloroplast genomes in gene arrangement and composition. Among these functional genes, 11 protein-coding genes and 6 tRNA genes contained introns (Supplementary Table S2), whereas, *ycf3* and *clpP* contained two introns. However, the *rps12* was a special trans-splicing gene which the 5′ exon of the gene located in the LSC region and the 3′ exon located in the IR region.

**Table 2:** Comparison of the complete chloroplast genome contents of four *Fagopyrum* species.

	*F. dibotrys*	*F. luojishanense*	*F. esculentum*	*F. tataricum*
GeneBank Number	KY275181	KY275182	EU254477	KM201427
Total Sequence Length	159.320	159.265	159,599	159,272
Large Signal Copy (LSC)	84.422	84.431	84,888	84,398
Small Singal Copy (SSC)	13.264	13.094	13,343	13,292
Inverted Repeat Region (IR)	30.817	30.870	30,684	30,791
Total Number of Gene	114	114	114	114
Protein coding genes	81	81	81	81
tRna	29	29	29	29
rRna	4	4	4	4
GC%	37,9	37,8	38.0	37.9

In this paper, we updated the annotation on the chloroplast genome sequence of two wild buckwheat species after used the DOGMA program. Based on the gene function, the different functional genes were divided into three categories (Supplementary Table S2). The genes of the first category was participate in transcription and translation, a total of 60 genes, most of them were tRNA including RNA polymerase, ribosomal RNA, ribosomal proteins products and so on. The second category was photosynthesis related genes, a total of 47 genes, including rubisco genes, genes participate in electron transfer of photosynthesis and NADPH dehydrogenase genes. The third category was amino acids and fatty acids biosynthesis related genes, including one pseudogenes and two unknown function genes.

### Sequence Divergence Hotspot Regions Analysis

**Figure 3:**
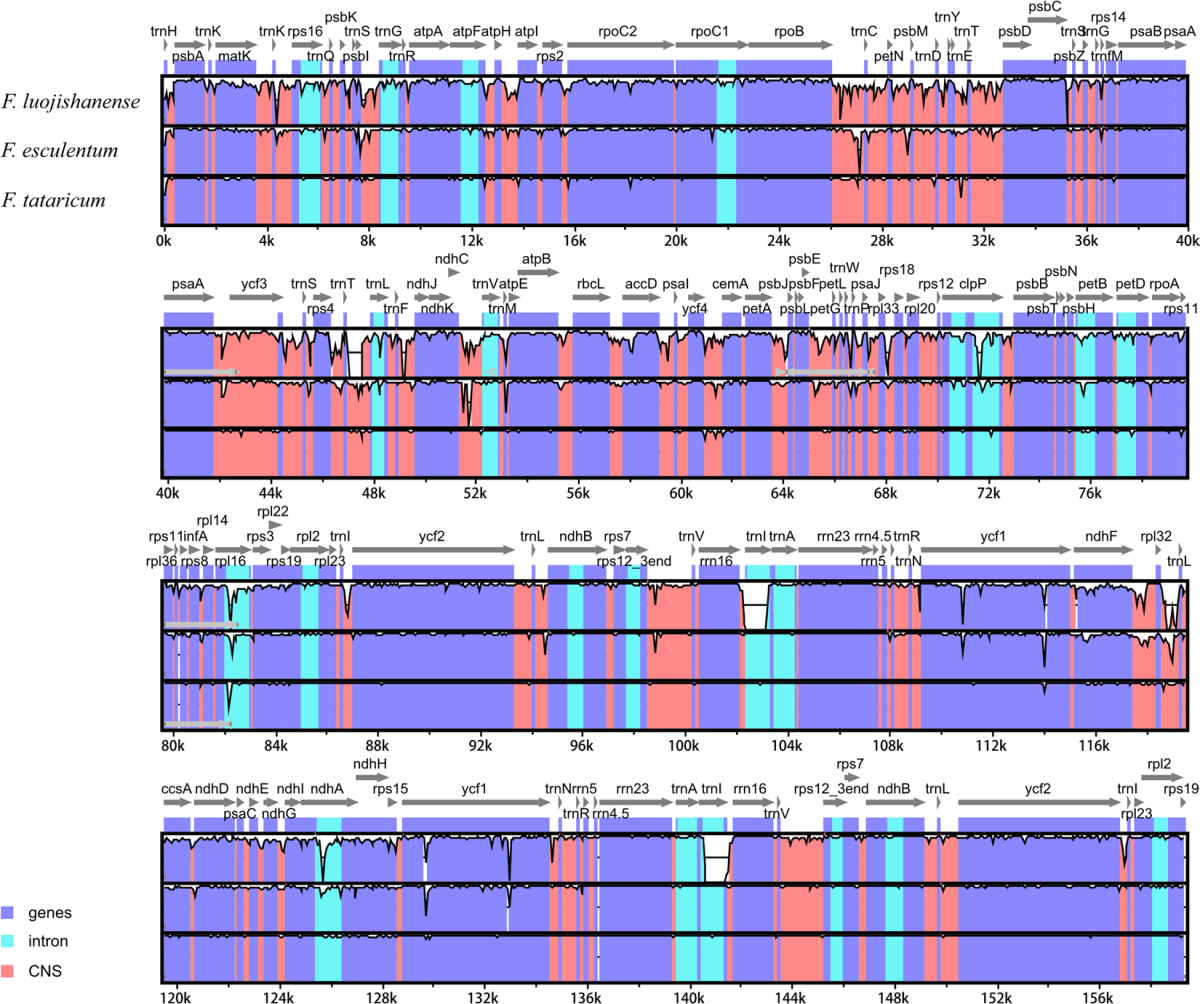
Sequence identity plots among the four *Fagopyrum* chloroplast genomes by using mVISTA. The y-axis represents identity ranging from 50% to 100%.

**Figure 4:**
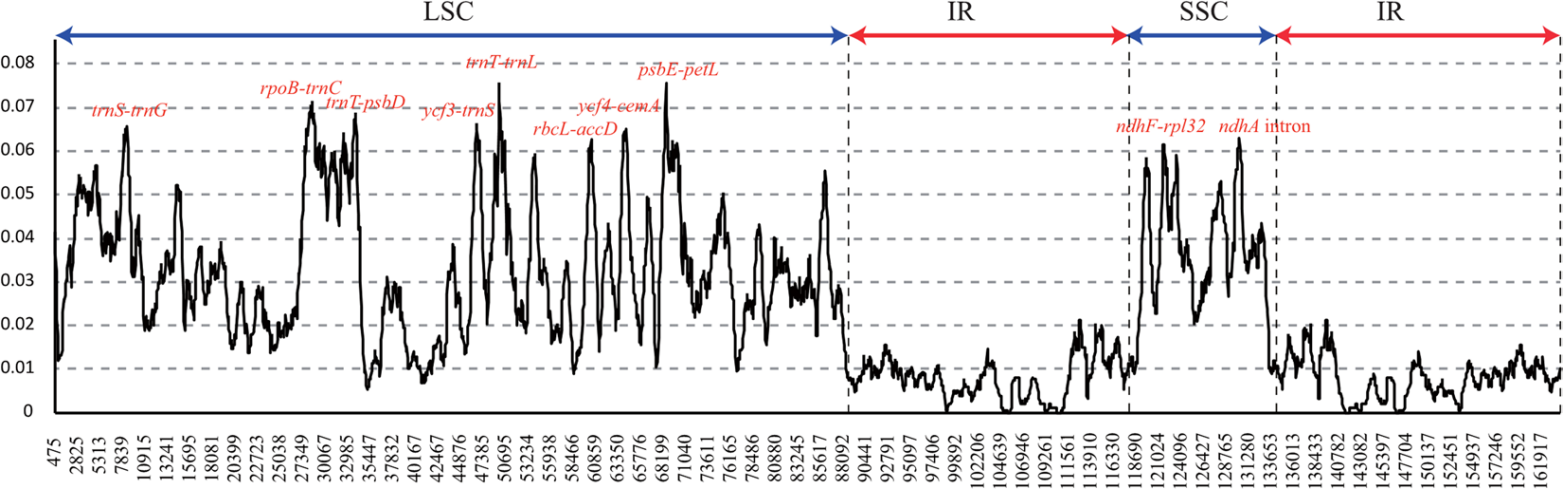
Sliding window analysis of the four *Fagopyrum* chloroplast genome sequences. The Y-axis represents nucleotide diversity of each window, while the X-axis represents position of the midpoint.

The mVISTA program was used to perform a sequence identity analysis and elucidate the level of sequence divergence after compared the complete chloroplast genomes of the four *Fagopyrum* species. The results were revealed in Fig. 3, and the alignment showed high sequence similarity which suggested that the four chloroplast genomes were conserved. At the same time, we also used the DnaSP 5.0 software to elucidate the sequence divergence, which shown that the nucleotide diversity (π) was 0.02372 among the chloroplast genomes of *Fagopyrum* species. After sequence divergence analysis across the four buckwheat chloroplast genomes, the total number of mutations (Eta) was 7152, and the number of polymorphic (segregating) sites (S) was 7001. The theta (per site) from S (Theta-W) was 0.02472 and the theta (per site) from Eta was 0.02525 while the theta (per sequence) from S was 3818.727. Meanwhile, the Theta (per sequence) from S was 3818.727. The sliding window of DnaSp 5.0 software was displayed in Fig. 4, ten highly variable regions were located precisely, which the nucleotide diversity (π) was higher than 0.06, including *trnS-trnG*, *rpoB-trnC*, *trnT-psbD*, *trnT-trnL*, *rbcL-accD*, *ycf4-cemA*, *psbE-petL*, *ndhF-rpl32*, *ndhA* and *ycf3-trnS*. And the *psbE-petL* regain has a highest Pi value which is 0.07563 (Supplementary Table S3). Eight of these regions located at the LSC region, two of them located in the SSC region but no one in the IR region. After that, to validate the ten highly variable regions, we also amplified the fragments of these regions. Four *Fagopyrum* plants were chosen to test the discriminatory powers of these high variable sequences. The result was shown in Supplementary Figure S1. All the bands in different lanes could been seen, which means these ten variable regions could be used as new molecular markers for phylogeny analysis in *Fagopyrum* species.

### Numbers and Pattern of SNP Mutations

We investigated the SNP mutations of the four chloroplast genomes, which were the most abundant type of mutations in the genomes. The numbers of nucleotide substitutions in four complete chloroplast genomes was shown in Table 3. We calculated the numbers of nucleotide substitutions between every two species. The number between *F*. *luojishanense* and other species was huge, which was 5940, 6260, 5992 between *F*. *dibotrys*, *F*. *esculentum* and *F*. *tataricum* respectively. In addition, we only found 317 nucleotide substitutions between *F*. *dibotrys* and *F*. *tataricum*, which was the smallest number across all the chloroplast genomes of the four species.

**Table 3:** Numbers of nucleotide substitutions in four complete chloroplast genomes.

	*F. dibotrys*	*F. luojishanense*	*F. esculentum*
*F. dibotrys*			
*F. luojishanense*	5940		
*F. esculentum*	2036	6260	
*F. tataricum*	317	5992	2105

### SSRs Polymorphisms

We used Perl script MISA to detect the SSRs mutations sites. SSRs consist of mononucleotide (A/T) repeats, dinucleotide (AT/TA) repeats, trinucleotide (CTT/AAG) repeats and tetranucleotide (GTCT/AATG/AATA) repeat were detected among the four *Fagopyrum* cp genomes. The number of SSRs in chloroplast genomes is different among the four *Fagopyrum* species which was shown in Table 4. The number of SSRs in *F*. *dibotrys* and *F*. *luojishanense* chloroplast genomes was 48 and 61, while the number of SSRs in *F*. *esculentum* and *F*. *tataricum* chloroplast genomes was 57 and 49 respectively. The mono-, di-, trin-, tetra-, penta-, and hexanucleotide SSRs were all counted among the four *Fagopyrum* chloroplast genomes. Two pentanucleotide repeats SSRs were found in *F*. *dibotrys* cp genome, and one pentanucleotide repeats SSRs were found in *F*. *tataricum* cp genome, and no hexanucleotide repeats were observed in these cp genomes. In *F*. *dibotrys* cp genomes, the mononucleotide repeats was the biggest with a portion of 58.33%, the dinucleotide, trinucleotide, tetranucleotide and pentanucleotide repeats were 11, 3, 4 and 2 respectively. In *F*. *luojishanense* chloroplast genomes, the mononucleotide repeats was highest with a portion of 62.30% among the species, the dinucleotide, trinucleotide, tetranucleotide and pentanucleotide repeats were 15, 4, 4 and 0 respectively. On the other hand, in the cultivated species, the mononucleotide repeats was the biggest with a portion of 57.89% while the dinucleotide, trinucleotide, tetranucleotide and pentanucleotide repeats were 16, 4, 4 and 0 in *F*. *esculentum*. At last, in *F*. *tataricum* cp genomes, the mononucleotide repeats was also the biggest with a portion of 61.22%, the dinucleotide, trinucleotide, tetranucleotide and pentanucleotide repeats were 11, 3, 4 and 1 respectively. Normally, the SSRs in cp genome were dominated by A/T mononucleotide repeats. From our results, the A/T repeat unit was the most abundant which has a particular repeat numbers of 10–15, and most repeats were consist of A or T.

**Table 4:** Types and number of SSRs in chloroplast genomes.

	*F. dibotrys*	*F. luojishanense*	*F. esculentum*	*F. tataricum*
mononucleotide repeats	28	38	33	30
dinucleotide repeats	11	15	16	11
trinucleotide repeats	3	4	4	3
tetranucleotide repeats	4	4	4	4
pentanucleotide repeats	2	0	0	1
hexanucleotide repeats	0	0	0	0
All types in complete cp genome	48	61	57	49

All SSRs with a length of at least 10 bp among the four *Fagopyrum* chloroplast genomes species were detected (Supplementary Table S4). Based on the results, we also found that many SSRs were observed in the same 14 locus among the four different *Fagopyrum* species. Most of these locations were intergenic, including *trnS-trnG*, *rpoB-trnC*, *trnS-psbZ*, *trnS-rps4*, *ndhC-trnV*, *rpl33-rps18*, *rpl32-trnL*, *ndhD-psaC*, the remaining five were located in protein-coding genes, which were *atpA*, *rpoC2*, *cemA*, *rpl22*, *ycf2* and *ndhD*. On the other hand, among the four *Fagopyrum* cp genomes sequences, SSRs mainly located in intergenic, following by genes and intron, and no SSRs were observed in the tRNAs and rRNAs. Most of those SSRs detected were located in LSC region, followed by IR and SSC regions. From our results, the IR reign speared by SSC contain the same number of nucleotide repeats because of concerted evolution in cp genome. In *F*. *dibotrys* cp genome, there were 16 SSRs in gene regions, 30 SSRs in intergenic regions and 2 SSRs in introns, which were *ycf3* intron and *petD* intron. Most of these SSRs were located in LSC region, which the number was 33, followed by IR and SSC regions. Meanwhile, 61 SSRs were detected in the chloroplast genome of *F*. *luojishanense*. These SSRs mainly located in intergenic spacer which have 43 SSRs, following by 11 in gene regions and 7 in introns regions. Of all these SSRs events, 46 SSRs were in the LSC region while 8 SSRs were in the IR region and 7 in the SSC region. At the same time, the SSRs in *F*. *esculentum* and *F*. *tataricum* were also detected used the same method. The number of SSRs in *F*. *esculentum* was little more than that in *F*. *tataricum*. But both of them mainly located in the LSC region, while 37 of 57 SSRs in the *F*. *esculentum* and 35 of 49 SSRs in the *F*. *tataricum* were in the LSC region.

### Repetitive Sequences Analysis

**Figure 5:**
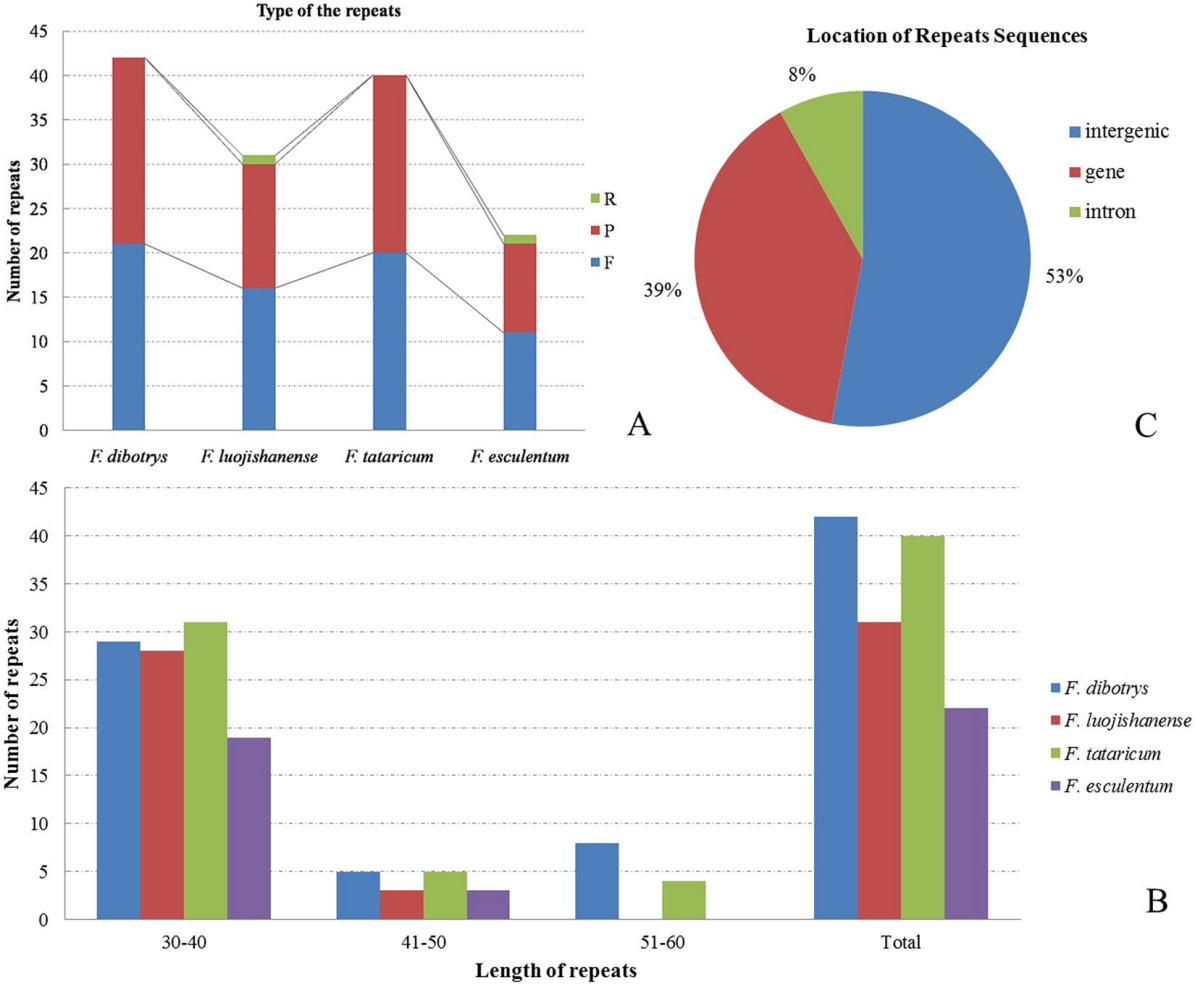
Numbers, repeat types and distributions of repeats in the four *Fagopyrum* chloroplast genomes. The letter F, P and R in Fig. 5A represent forward, palindromic and reverse repeats.

We used the REPuter to find all kinds of repeats in the cp genome sequences, the results of repetitive sequences analysis were shown in Fig. 5 with the criterion of copy size 30 bp or longer. A total of 135 repeats were detected in the four *Fagopyrum* cp genomes sequences, including forward, palindromic and reverse repeats, which abbreviated as letter F, P and R in Supplementary Table S5. Nevertheless, the reverse match repeats only been found in *F*. *luojishanense* and *F*. *esculentum* cp genomes. While the *F*. *dibotrys* and *F*. *tataricum* cp genomes only included two repeat types, forward and palindromic. We identified a total of 42 repeats in the *F*. *dibotrys* cp genomes and 31 repeats in the *F*. *luojishanense* cp genomes which including forward, reverse and palindromic repeats. At the same time, we also identified a total of 40 repeats in the *F*. *tataricum* chloroplast genomes and 22 repeats in the *F*. *esculentum* chloroplast genomes (Fig. 5A). The wild *Fagopyrum* species *F*. *luojishanense* possessed 16 forward repeats, 14 palindromic repeats and one reverse repeats, except for the other wild species *F*. *dibotrys* with 21 forward repeats and 21 palindromic repeats. On the other hand, we also detected 20 forward repeats and 20 palindromic repeats in *F*. *tataricum* chloroplast genomes while 11 forward repeats, 10 palindromic repeats and one reverse repeats in *F*. *esculentum* chloroplast genomes. The number of the forward repeats and palindromic repeats are basically the same, accounting for 68 forward repeats of the total repeats, and 65 palindromic repeats, following with two reverse repeats among the four cp genome sequences.

Most repeats sequences were detected in IR region, following by LSC region, and the SSC region also has a few repeats been found. The repeat length of the first part in four *Fagopyrum* cp genomes ranged from 30 to 59 bp. Most starting position of the first part of repetitive sequences were distributed in IR regions of *F*. *dibotrys* cp genomes. On the other hand, in *F*. *luojishanense* chloroplast genomes, most starting position of the first part of repetitive sequences were distributed in LSC region, only minority starting position of the first part of repetitive sequences were located in SSC regions (Supplementary Table S5). The lengths of most repeats in these four *Fagopyrum* cp genomes was shorter, mainly ranging from 30 to 40 bp (Fig. 5B), whereas the longer repeats, such as ranging from 51 to 60 bp, was also be found in the *F*. *dibotrys* and *F*. *esculentum*.

At the same time, we found the distribution of repeats are conserved among four different cp genomes. In this study, we investigated the location of these repeats. It was found that the majority repeats were located in intergenic spacer (53%) and gene coding regions (39%), while a minority of repeats was detected in intron (8%) (Fig. 5C). After that, we analysis the repeats deeply, and found there has many repeats shared in the same locus, which indicated that the repeats sequences could be detected in these locus among the four cp genomes. Here, we found nine locus speared in three different region, which all could detected repeats sequences among four *Fagopyrum* cp genomes. These locus were *pasA*, *pasB*, *psbI-trnS*, *psbC-trnS*, *ycf3* and *rbcL-accD* in LSC, flowing with *rrn4*.*5-rrn5*, *ycf1* in IR region and *ndhA* in SSC. At the same time, we also found the numbers, repeat types and distributions of the repeats between the cp genomes of *F*. *dibotrys* and *F*. *tataricum* were similar and conserved. From our results of highly divergent hotspot regions, it also showed that the repeat sequences were associated with the divergent regions of cp genomes, such as the *ndhA* and *rbcL-accD*. It implied the repeats sequences also could be used as genetic markers for phylogenetic studies.

### Phylogenetic Analysis

**Figure 6:**
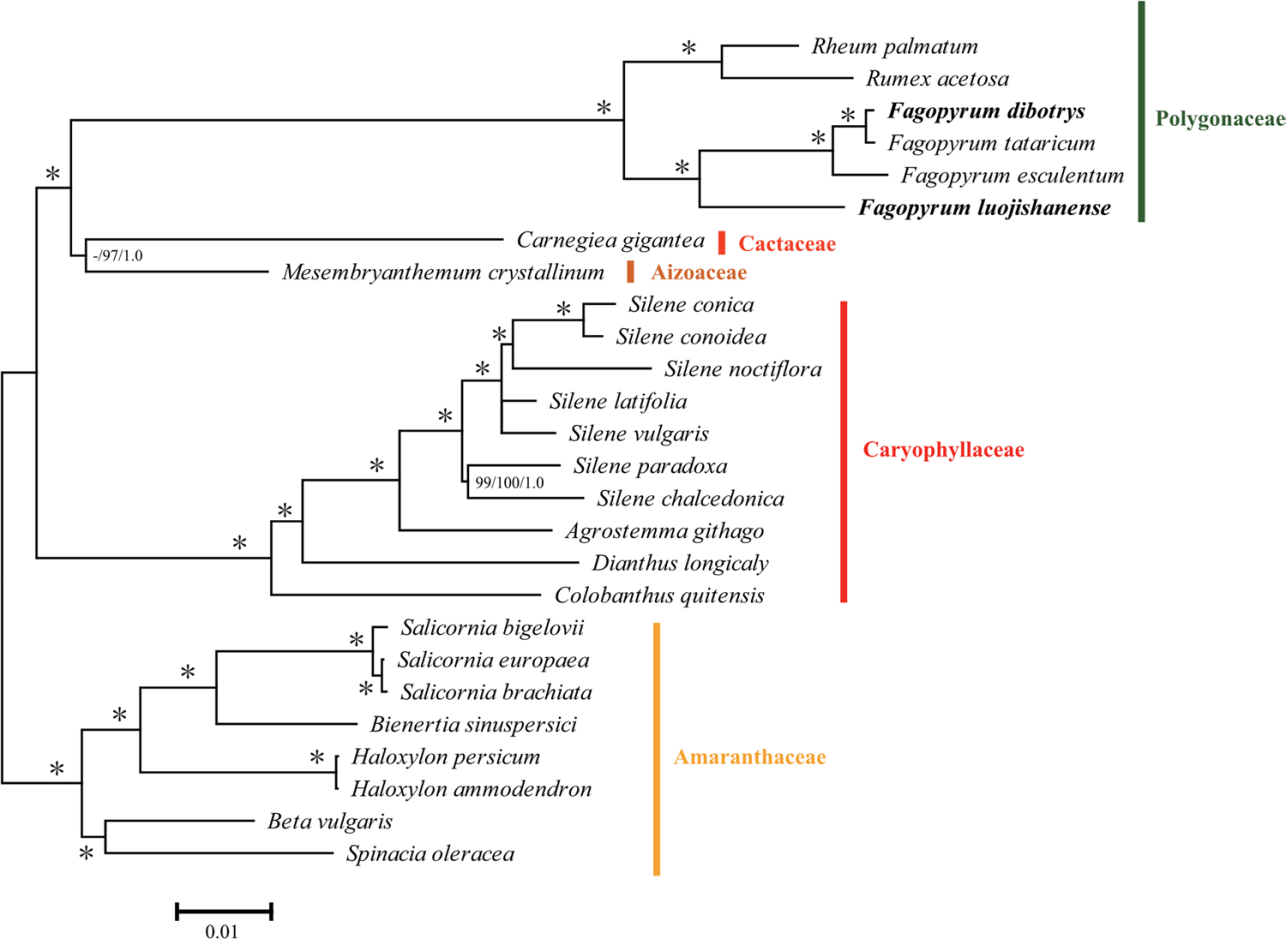
Phylogenetic relationships of the four *Fagopyrum* species inferred from MP/ML/BI analysis constructed by chloroplast genome. The numbers associated with each node are bootstrap support values, and the symbol * in the phylogenetic tree indicated that the support rate of branch is 100/100/1.0.

In the present studies, four complete chloroplast genomes of *Fagopyrum* species and 24 outgroups were used to construct the phylogenetic trees. These outgroups were shown in Supplementary Table S6, which came from five different families that contain all cp genomes gene data in the *Caryophyllales*. And we used three different methods MP/ML/BI to build a phylogenetic tree based on these gene data. Figure 6 showed the results of ML analysis, which was completely coincident with the phylogenetic tree that build by the MP and BI analysis. The symbol * in the phylogenetic tree indicated that the support rate of branch was 100/100/1.0. It was clear that all the species were classified into five big groups, and every species in each group came from the same families which confirmed these species came from five different families. The results showed that all the *Fagopyrum* species cluster together with much higher internal resolution, and they were classified into a big group with *Rheum palmatum* and *Rumexacetosa*. In this *Polygonaceae* group, *F*. *dibotrys*, *F*. *tataricum*, *F*. *esculentum* and *F*. *luojishanense* formed a subgroup, which was different with *Rheum palmatum* and *Rumexacetosa* subgroup. As the Fig. 6 illustrated, *F*. *dibotrys* was closer to *F*. *tataricum* than to *F*. *esculentum*. In addition, *F*. *dibotrys* was closer to *F*. *tataricum* and *F*. *esculentum* than to *F*. *luojishanense*. The results suggested that *F*. *dibotrys* was more closely related to *F*. *tataricum*.

## Discussion

The morphology data of different species could reflect the difference among species. Besides, the morphology data and agronomy data like amino acids content have already been used to determine the differences between species. From the result, two wild buckwheat species have been characterized by leaf shape, petioles and flower type, on the basis of morphological characteristics, *F*. *loujishanenes* was distinguishable from the two cultivated species easily. Genetically, *F*. *loujishanenes* is also quite distant to the other species of *Fagopyrum*. *F*. *dibotrys* has many similarities with *F*. *tataricum* and *F*. *esculentum* in morphological characteristics, especially with petal, leaf, inflorescence type and seed color. Although the *F*. *dibotrys* and two cultivated species are genetically similar, they differ considerably in habit and gross morphology. Sometimes, the morphology of species in genus is hard to distinguish different species accurately. On the other hand, the PCA result based on the means of morphology profiles and agronomy data also cannot well determine the differences among the four buckwheat species. In the PCA result, wild buckwheat *F*. *luojishanense* was obviously distinguished from other three buckwheat, but it is still hard to discriminated the other three buckwheat species clearly. Therefore, the divisions and the evolutionary relationships within *Fagopyrum* species need further investigating and searching more evidences. Combined evidence from morphology characteristics and cp DNA has been served as a powerful tool in hybrid studies^27^. On the other hand, it has been reported that the cp DNA genomes independent from the mechanism of cytoplasmic in the study of the modified flower type with the chloroplast DNA^28^. Because of the parallel and convergent evolution of morphological characteristics, the data of morphological analysis maybe internally inconsistent. Only based on morphological characteristics, we could not conclusive with distinction and identification the hybrids, wild relative species and paternal species^29^.

The chloroplast genomes normally have a circular structure which ranging from 115 to 165 kb in length, and it is composed from a large single copy (LSC) region, a small single copy (SSC) region and two copies of inverted repeat (IR) regions^30^. Both chloroplast genomes of the two *Fagopyrum* species showed a typical circular structure, which consisted of two copies of IR regions separated by the LSC region and the SSC region. The chloroplast genomes contain much genetic information, which could be discovered through comparative analysis of complete chloroplast genome sequences. From the results, the sequence divergence of both IR regions is lower than LSC and SSC regions while it was also occurred in many plants which have been reported before. And it probably due to the gene conversion between IR sequences^31^.

The molecular markers such as high variable sequences, SSRs and SNPs are useful tools in research. Comparative analysis of complete chloroplast genome sequences of five *Camellia* species identified 15 molecular markers which have over 1.5% sequence divergences. After that, these high divergent sequences were used to phylogenetic analysis and species identification^32^. Our analysis data could serve to enrich the resource of *Fagopyrum* in systematic, molecular phylogenetic and genetic breeding studies. The mutation region in the cp genome was not random, and these mutational dynamics created the highly variable regions in the genome^33^. Ten highly variable regions been detected, including *trnS-trnG*, *rpoB-trnC*, *trnT-psbD*, *ycf3-trnS*, *trnT-trnL*, *rbcL-accD*, *ycf4-cemA*, *psbE-petL*, *ndhF-rpl32* and *ndhA* intron. As our results showed, most of them occurred in the LSC and SSC regions but not IR regions. Among these variable regions, the largest was located in the LSC region within the intergenic sequence *trnT-trnL*, and other two large variable regions were also found within *psbE-petL* and *rpoB-trnC* in the LSC region. Many highly variable regions have been identified in intergenic spacers, including *ndhF-rpl32*, *trnE-trnT*, *rpl32-trnL*, *trnQ-rps16* and some other protein coding genes, like *rpl20*, *ycf1*, *ycf15* and *accD* ^34^. Among these regions, the highly variable regions *rpoB-trnC*, *psbE-petL*, *trnT-psbD* and *rbcL-accD* have been reported in seed plants before, these regions showed very high nucleotide diversity per site (p-values) after across comparison^35^. At the same time, the *ycf4-cemA* also has been reported recently after comparative analysis of whole plastid genomes from the *Apiales* ^36^, while the *ndhF-rpl32* and *trnT-trnL* also have been reported as a molecular maker for phylogeny analysis in *Machilus* ^37^ and *Lupinus* ^38^. Especially the *trnS-trnG* region, it has been used as the molecular maker for phylogenetic relationship analysis^39^ and solving origin problems^40^ in many species, such as *Solanaceae* ^41^, *Bromeliacea* ^42^ and *Lamioideae* ^43^.

However, the *ycf3-trnS* and *ndhA* intron seem to be especially variable in *Fagopyrum*, which were rarely reported highly variable regions before. For now, the most important is these highly variable regions have not been used as potential molecular markers to investigate the phylogenetic relationships and identification of *Fagopyrum* species, except the *rpoB-trnC* ^44^, *rbcL-accD* ^8^ and *ndhF-rpl32* ^45^. In contrast, the *matK* and *trnK* which in the chloroplast genome used for phylogenetic analysis of wild *Fagopyrum* species previously was not found to be highly variable^10, 11^. And most of these variable sequence regions had higher variation percentages than *rbcL-accD*, which has been used for interspecific relationships study before^8^. All of these highly variable regions are better to use for phylogenetic analysis in *Fagopyrum* at the species level. Because of the abundant germplasms resources and complex evolutionary issues, the taxonomy of *Fagopyrum* is still difficult to assess, especially the taxonomy status of wild species. Therefore, we believe these ten highly variable regions could provide abundant information for developing molecular markers, phylogenetic analysis and identification of *Fagopyrum* species.

There are many forms of SSRs in the genome, and the copy numbers of SSRs was different between species. Compared to other neutral DNA regions, SSRs usually have higher mutation rate because of slipped DNA strands, which could be used as potential genetic markers for assays detecting polymorphisms at population-level, phylogenetic relationships studies among species and plant ecological studies^46^. At the same time, the SSRs polymorphisms study has been used to investigate the evolutionary relationships among closely related species recently, which has the advantages of low cost but high precision^47^. From the results, the most abundant one was the mononucleotide repeats, followed by the di-, trin-, tetra-, pentanucleotide. All in all, the number of trinucleotide repeats and tetranucleotide repeats is substantially the same, and the number of pentanucleotide was very small across the chloroplast genomes. At the same time, no hexanucleotide SSRs was detected among the chloroplast genomes. These SSRs identified in our study could useful in phylogenetic and evolutionary studies as well as they were in *Cocos nucifera* ^48^, *Pyrus pyrifolia* ^49^ and *Elodea Canadensis* ^50^.

On the other hand, large and complex repeat sequences possibly related with the chloroplast genomes sequence divergence and rearrangement^51^. It has been suggested that the repeat sequences played an important role in chloroplast genomes variation and sequence rearranging because of the recombination^46^ and slipped-strand mispairing^52^. From our results, it also shown that there are connections between divergent regions of chloroplast genome and various repeat sequences. And most repeat sequences of *F*. *luojishanense* detected in the LSC and SSC regions which have higher sequence divergence than IR regions. Based on the results of highly divergent regions, it also showed that the repeat sequences were associated with the divergent regions of cp genomes, such as the *ndhA* and *rbcL-accD*. Interestingly, in *F*. *dibotrys* chloroplast genome sequences, most repeat sequences were located at the IR regions which was the same as the distribution of most repeat sequences of *F*. *tataricum*. This fact also could explain *F*. *dibotrys* has a closer phylogenetic relationship with *F*. *tataricum*. All in all, these repeats maybe further serve as potential genetic markers for phylogenetic studies on *Fagopyrum* species.

The single nucleotide polymorphism has been proven to have significant potential for genome structure analysis and species identification. From the results, the smallest number of nucleotide substitutions is 317, which was detected between *F*. *dibotrys* and *F*. *tataricum* chloroplast genome. It indicated the nucleotide substitution events between the chloroplast genome of *F*. *dibotrys* and *F*. *tataricum* are less than species of ginseng, potato, and orange^53, 54^. But most nucleotide substitutions events which detected between two *Fagopyrum* species was larger than 2000, especially the nucleotide substitutions between *F*. *luojishanense* and the two cultivated species. The nucleotide substitution events detected between *F*. *luojishanense* and *F*. *esculentum* was 6260 while the nucleotide substitution events detected between *F*. *luojishanense* and *F*. *tataricum* was 5992, which indicated that the variations in wild species are much higher than in cultivated species.

The chloroplast genome sequences have been used for the phylogenetic studies in many species successfully^55–57^. From the results, these phylogenetic analysis identify major geographic groups similar to which have been researched using the ITS and *matK* for phylogeny study before. But in our study, there are much higher internal resolution with a high level of bootstrap values, which reflecting the higher number of base substitutions between wild and cultivated species. It indicated that the cultivated species have obviously evolutionary trends compared with the wild species. The phylogenetic tree based on the analysis of chloroplast genome has highly supported nodes, which nearly resolved the phylogenetic problems between wild and cultivated buckwheat. In outline of classification of magnoliophyta, Cronquist suggested the *Fagopyrum* belongs to family *Polygonaceae*, order *Polygonales* ^58^. However, in the classification of APG III, the *Fagopyrum* belongs to family *Polygonaceae*, order *Caryophyllales*. Our results showed that APG III taxonomic system of *Fagopyrum* is reasonable on the whole and phylogenetic relationships within buckwheat species^59^. At the same time, our phylogeny inferred from complete chloroplast genome sequences also showed the *F*. *dibotrys* is closer to *F*. *tataricum* than to *F*. *esculentum* ^10^. Phylogenetic analysis of chloroplast sequences and morphology dates all indicate a unique position of *F*. *loujishanenes*. The positions of two wild species *F*. *loujishanenes* and *F*. *dibotrys* were consistent, and the two species both have high bootstrap values (<95%). We found convincing evidence for the phylogenetic relationship of *F*. *loujishanenes* and *F*. *dibotrys* from *Fagopyrum*. All in all, our results suggest that the chloroplast genome data could resolve the phylogenetic relationships of *Fagopyrum* effectively. Meanwhile, the evolutionary relationships of *Fagopyrum* genus also need further investigation and more complete evidences.

## Materials and Methods

### Morphological analysis

Plant materials were cultivated in green house of Sichuan Agricultural University. The young single seedlings of four different buckwheat species were collected for the morphological comparison. The details of plant materials used were observed and measured. To analysis *Fagopyrum* species based on morphological differences, we mainly focus on the plant type, stems, leaf, inflorescence and seeds. As food and medicinal raw materials, we also focus on the agronomic characteristics of these buckwheat. The methods of observation and measurement are shown in Table 5. The analysis data were checked and measured for 3 plants of each species. All observation data were designed in completely randomized block design. In this section, we also used four morphology data including plant height, stem thick, leaf long and leaf wide, as well as four agronomy data which were thousand grain weight, protein content, flavonoids content and amino acid content from four different species to reflect the differences of these buckwheat based on PCA. The PCA was used to reduce the dimensionality of the morphology and agronomy data for further determined and evaluate the difference of four *Fagopyrum* species. The statistical analysis were performed using IBM SPSS Statistics software version 16.0 (SPSS Inc., Chicago, IL, USA).

**Table 5:** Methods of observation and measurement.

Item	Observation and Measurement
Plant type	We observed the plant type in sampling process after two month cultivated, and the plant type was divided into three types, including erect, semi-erect and grovel type. The plant height (the base of the stem to the top) was measured and calculated.
Stem	The color of plant was also observed in mature period, and the plant color was divided into three types, red-brown, red and green. The plant stem diameter was measured at the base of the main stem using slide caliper rule. The number of stem nodes was calculated using magnifier.
Leaf	On the other hand, we observed the shape of leaves and petioles using magnifier.
Flower	We observed the inflorescence type, perianth, length of pistil and stamens during the florescence.
Seed	We collected the mature seeds and the following indexes were observed, including seeds color, seeds shape and seeds winged.
Thousand grain weight	Count out one thousand seeds randomly, measure the weight then take the average.
Protein content	Weigh 10 g seeds, the shells of seeds were removed and smashed into powder, and the powder was filtered through a mesh screen (aperture: 0.25 mm) for determination. The content of protein was determined by Kjeldahl’s method.
Amino acid content	The content of amino acid was determined by Hitachi 835-50 Automatic Amino Acid Analyzer.
Flavonoids in seeds	Weigh 1 g of each sample, heated and extracted repeatedly with methanol. 0.5 ml of extracted solution was taken into a test tube, and 10 ml of aluminum chloride in anhydrous methanol was added. After blending, the flavonoids content was determined with colorimetry.

### DNA Extraction, Sequencing, Assembly

The young leaves of *F*. *dibotrys* and *F*. *luojichanese* from single seedlings were sampled for DNA Extraction. Total genomic DNA of buckwheat was extracted using the modified CTAB protocol from 100 mg fresh leaves^60^. For all the *Fagopyrum* species in this paper, total genomic DNA was broken by ultrasound, yielded fragments of 300–500 bp in length, and the fragmentation quality was checked using Bioanalyzer 2100 (Agilent Technologies). The library of 400 bp DNAs was constructed using the NEBNext Ultra™ DNA Library Prep Kit (Illumina, San Diego, California, USA), and the genomic DNAs of the four *Fagopyrum* species were sequenced using HiSeq. 4000 PE150 (Illumina Inc.). For each of the species, the High-throughput sequencing dates were spliced using SPAdes 3.6.1^61^ and SOAPdenovo2^62^. The high quality reads were obtained using the CLC-quality trim tool, and the contig of the chloroplast genome was selected using the Blast program^63^. The contig of the chloroplast genome was assembled using Sequencher 4.10 (http://www.genecodes.com) with default parameters and the gaps of sequence found in the splicing were amplified with PCR-based conventional Sanger sequencing using ABI 3730. The specific primers at both ends of the gap of the four sampled species were designed for PCR until the chloroplast genome sequence is complete. The PCR program is 94 °C for 4 min followed by 34 cycles of 94 °C for 30 s, 55 °C for 30 s, 72 °C for 1.5 min, and 72° for 10 min. After that, all reads were mapped to the spliced chloroplast genome sequence using Geneious 8.1^64^ to avoid assembly errors and proofread the contig is correct. Finally, we obtained two wild buckwheat high quality complete chloroplast genome sequences.

### Chloroplast Genome Annotation

The gene annotation of the four chloroplast genomes was performed using DOGMA^65^, and the positions of coding genes, transfer RNAs and ribosomal RNAs were searched and identified by BLASTX and BLASTN. The potential coding gene promoter/terminator as well as the intron/exon boundaries were manually corrected and selected by compared to homologous genes of other sequenced chloroplast genomes. Because of the limitations of BLAST, some of the short exon regions (6–9 nt) cannot be annotated with the DOGMA, such as *rps16*, *petB*, *petD* and some intron and exon boundaries are not well recognized in the chloroplast genome annotation process. For these genes, we accurately adjusted and annotated them according to other published genomes. The circular genome maps of *F*. *dibotrys* and *F*. *luojishanense* were drawn by Organellar Genome DRAW (http://ogdraw.mpimp-golm.mpg.de/index.shtml)^66^.

### Sequence Divergence Hotspot Regions Analysis

Comparison among the four *Fagopyrum* chloroplast genomes was performed using the mVISTA program^67^ to show the interspecific variation. First of all, the four chloroplast genomes were aligned by MAFFT v5^68^ with the default parameters set and then the sequences were manually adjusted using Se-al (http://tree.bio.ed.ac.uk/software/seal.html) where necessary. The principle of multiple alignments was pulling open for the inversion of the sequence, in case the wrong data polymorphism occurs. The variation sites and sequence polymorphisms across the four chloroplast genomes were analyzed using DnaSp 5.0^69^ after aligned. We used the Sliding Window of DnaSp 5.0 software for investigating and analyzing the hyper-mutation fragment all over the sequenced *Fagopyrum* chloroplast genomes, and the window length was set to 800 bp and step size was set as 50 bp. The LSC, SSC and IR regions of the four species were calculated also using the DnaSP v5.0 software. The primer designed based on the sequence of these four *Fagopyrum* species was showed in the Supplementary Table S7. PCR amplification to validate these hotspot regions carried out in 25 µL volumes containing 9.5 µL Taq Mix (Takara Bio, Shiga, Japan), 12.5 µL ddH_2_O, 1 µL forward primer, 1 µL reverse primer and 1 µL genomic DNA. The PCR protocol as follow: 94 °C 5 min; 94 °C 40 s, 58 °C 45 s, 72 °C 1 min for 32 cycles, 72 °C 10 min. After that, PCR products were determined through electrophoresis on ethidium bromide-stained agarose gels in 1X Tris Borate EDTA (TBE), and amplification profiles were photographed using Gel Doc™ XR System (Bio-Rad Inc.).

### SSRs and Repeat sequence characterization

For the SSRs detection, the microsatellites were analyzed using the Perl script MISA^70^, and the SSRs parameters were defined as follow, thresholds of ten repeat units for mononucleotide SSRs, five repeat units for dinucleotide SSRs, four repeat units for trinucleotide SSRs, and three repeat units for tetra-, penta-, and hexanucleotide SSRs. We used the REPuter to find all kinds of repeats in the chloroplast genome sequences (http://bibiserv.techfak.uni-bielefeld.de/reputer), including forward match repeats, reverse match repeats, complement match repeats and palindromic match repeats. The minimal repeat size was 30 bp and the Maximum Computed Repeats was 60 bp^71^. Meanwhile, the similarity between two repeat copies was 90% at least, and the gap size between repeats had a maximum length of 3 kb.

### Phylogenetic Analysis

Phylogenetic analysis was conducted based on the gene sequences of *Fagopyrum* and other taxa of basal dicotyledonous. In this study, we used 26 species from 5 different families, including 6 species in the *Polygonaceae* for investigating the evolution of *Fagopyrum*. The 20 species (*Sileneconica*, *Carnegiea gigantean*, *Salicornia brachiate* and so on) were used as outgroups. The chloroplast genome and the nucleotide sequence data were obtained from NCBI. The phylogenetic trees which based on maximum likelihood analysis were performed using RAxML v7.2.8^72^, and the bootstrap replicates were 1000. The phylogenetic trees which based on maximum parsimony analysis were conducted using PAUP v4b10^73^, and 1000 random addition sequenceswere used for Heuristic search. The phylogenetic trees which based on Bayesian inference was performed using MrBayes v3.1.2^74^. Markov chain Monte Carlo simulations run twice for 2 million generations independently, and the remaining trees were used to construct a majority-rule consensus tree after the first 25% of trees was discarded.

## Electronic supplementary material

Supplementary Information

Supplementary Table S1

Supplementary Table S2

Supplementary Table S3

Supplementary Table S4

Supplementary Table S5

Supplementary Table S6

Supplementary Table S7

Supplementary Figure S1
