# Supplementary material for: Comparative Analysis of Four Buckwheat Species Based on Morphology and Complete Chloroplast Genome Sequences
Source: Sci Rep. 2017 Jul 26;7:6514. doi: 10.1038/s41598-017-06638-6 (PMC5529468; doi:10.1038/s41598-017-06638-6)
Supplement: Supplementary file 1 — Supplementary Information [file 41598_2017_6638_MOESM1_ESM.doc]

**Supplementary Information**

**Title of the manuscript**

Comparative Analysis of Four Buckwheat Species Based on Morphology and Complete Chloroplast Genome Sequences

**Author list**

CHENG-LONG WANG^1#^, MENG-QI DING^1,4#^, CHEN-YAN ZOU^1^, XUE-MEI ZHU^2^, YU TANG^3^, MEI-LIANG ZHOU^4*^ & JI-RONG SHAO^1*^

*^1^School of Life Sciences, Sichuan Agricultural University, Yaan, Sichuan 625014, China*

*^2^School of Resources and Environment, Sichuan Agricultural University, Chengdu, Sichuan 611130, China*

*^3^Department of Tourism Culture, Sichuan Higher Institute of Cuisine, Chengdu, Sichuan, 610072, China*

*^4^Biotechnology Research Institute, Chinese Academy of Agricultural Sciences, Beijing 100081, China*

*#* CHENG-LONG WANG *and* MENG-QI DING *contributed equally to this article.*

**Author for correspondence: e-mail: shaojr007@163.com* (JI-RONG SHAO)

*zhoumeiliang@caas.cn* (MEI-LIANG ZHOU)

Supplementary Table S1 Agronomic characteristics data of different buckwheat

Supplementary Table S2 A list of genes found in the plastid genome of *Fagopyrum* sp.

Supplementary Table S3 The sliding window of DnaSp

Supplementary Table S4 The detail of simple sequence repeats in the *Fagopyrum* chloroplast genomes

Supplementary Table S5 Analysis of repeated sequences in the four *Fagopyrum* chloroplast genomes

Supplementary Table S6 Date of the outgroups for the construction of phylogenetic trees

Supplementary Table S7 The primer designed for highly variable regions amplification

Supplementary Figure S1 Validation of hotspot regions by PCR
