# Supplementary figures and images for: Comparative Analysis of Four Buckwheat Species Based on Morphology and Complete Chloroplast Genome Sequences
Source: Sci Rep. 2017 Jul 26;7:6514. doi: 10.1038/s41598-017-06638-6 (PMC5529468; doi:10.1038/s41598-017-06638-6)

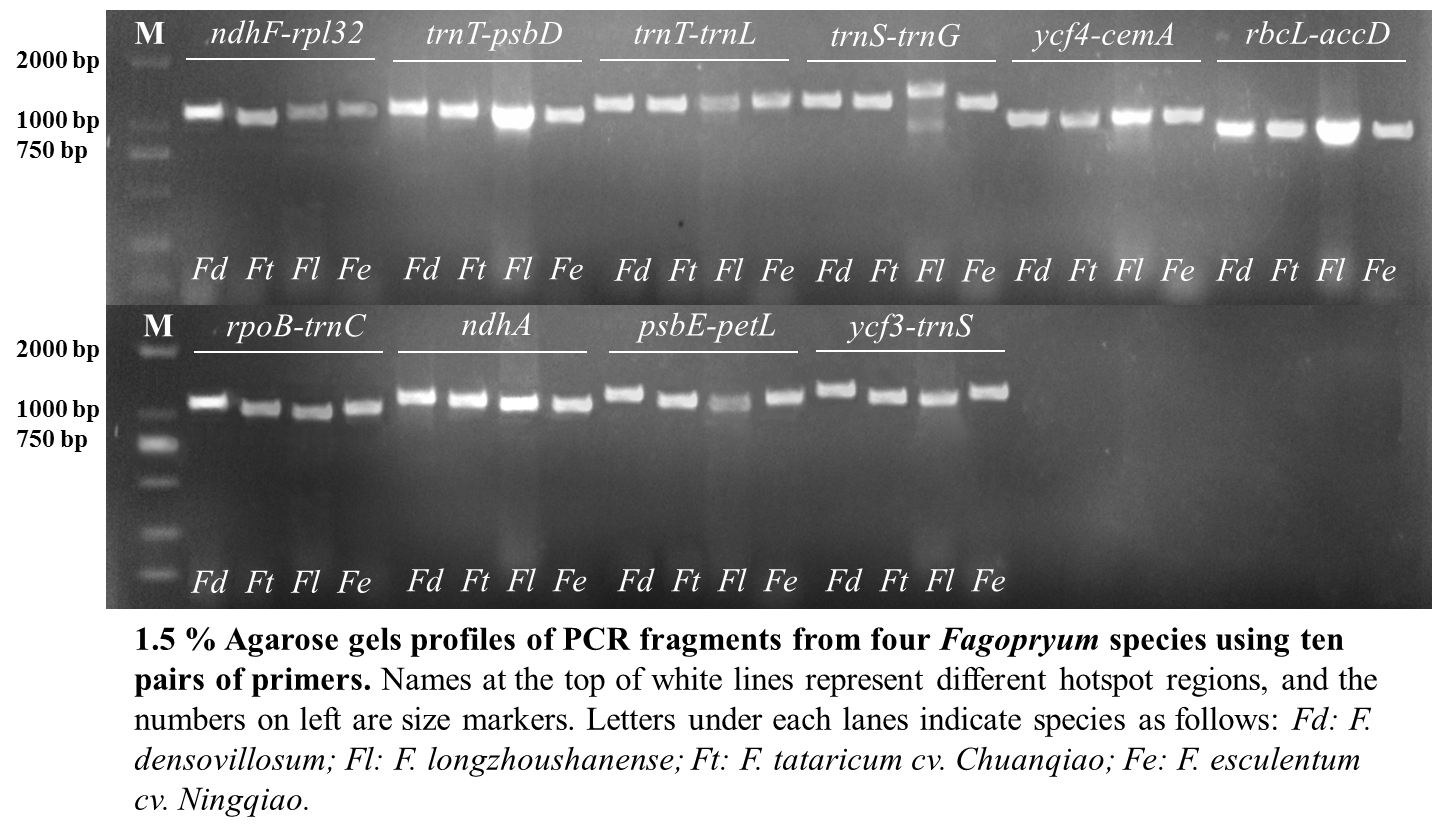
**Supplementary Figure S1 Validation of hotspot regions by PCR**

Supplement: Supplementary file 9 — Supplementary Figure S1 [file 41598_2017_6638_MOESM9_ESM.doc]
